# Recombinant Human Endostatin Suppresses Mouse Osteoclast Formation by Inhibiting the NF-κB and MAPKs Signaling Pathways

**DOI:** 10.3389/fphar.2016.00145

**Published:** 2016-06-01

**Authors:** Nong Chen, Ru-Feng Gao, Feng-Lai Yuan, Ming-Dong Zhao

**Affiliations:** ^1^Department of Orthopaedic Surgery, Zhongshan Hospital, Qingpu Branch, Fudan UniversityShanghai, China; ^2^Department of Orthopaedics and Central Laboratory, The Third Hospital Affiliated to Nantong UniversityWuxi, China; ^3^Department of Orthopaedics, Jinshan Hospital, Fudan UniversityShanghai, China

**Keywords:** recombinant human endostatin, osteoclast formation, NF-κB, MAPKs, rheumatoid arthritis, bone destruction

## Abstract

Rheumatoid arthritis is an autoimmune disease characterized by synovial hyperplasia and progressive joint destruction. As reported previously, recombinant human endostatin (rhEndostatin) is associated with inhibition of joint bone destruction present in rat adjuvant-induced arthritis; however, the effect of rhEndostatin on bone destruction is not known. This study was designed to assess the inhibitory effect and mechanisms of rhEndostatin on formation and function of osteoclasts *in vitro*, and to gain insight into the mechanism underlying the inhibitory effect of bone destruction. Bone marrow-derived macrophages isolated from BALB/c mice were stimulated with receptor activator of NF-κB ligand (RANKL) and macrophage colony-stimulating factor to establish osteoclast formation. Osteoclast formation was determined by TRAP staining. Cell viability of BMMs affected by rhEndostatin was determined using a MTT assay. Bone resorption was examined with a bone resorption pits assay. The expression of osteoclast-specific markers was analyzed using quantitative real-time PCR. The related signaling pathways were examined using a Luciferase reporter assay and western blot analysis. Indeed, rhEndostatin showed a significant reduction in the number of osteoclast-like cells and early-stage bone resorption. Moreover, molecular analysis demonstrated that rhEndostatin attenuated RANKL-induced NF-κB signaling by inhibiting the phosphorylation of IκBα and NF-κB p65 nuclear translocation. Furthermore, rhEndostatin significantly inhibited the activation of RANKL-dependent mitogen-activated protein kinases, such as ERK1/2, JNK, and p38. Hence, we demonstrated for the first time that preventing the formation and function of osteoclasts is an important anti-bone destruction mechanism of rhEndostatin, which might be useful in the prevention and treatment of bone destruction in RA.

## Introduction

Severe destruction of the adjacent cartilage and bone is the pathologic hallmark of rheumatoid arthritis (RA), and ultimately affects one’s ability to do physical activities, thereby reducing the quality of life (Ochi et al., 2007; Wei et al., 2013). It has been shown that osteoclasts play a critical role in local bone erosion in RA joints (Gravallese, 2002). In RA, numerous multinucleated osteoclast-like cells are present at sites of bone erosion, followed by degradation of the bone matrix (Pettit et al., 2006). In contrast, it has been demonstrated that bone erosion does not appear in osteoclast-deficient mice in arthritis models (Pettit et al., 2001). Moreover, high receptor activator of NF-κB ligand (RANKL), an essential factor for osteoclast formation, has been detected specifically in the synovium of RA patients (Lubberts et al., 2002). Therefore, osteoclasts are considered to be a type of critical target cell for inhibiting bone erosion in patients with RA.

Osteoclasts are large, multinucleated cells derived from the fusion of monocyte-macrophage lineage precursors. Osteoclasts play a key role in skeletal development and maintenance (Hayashibara et al., 2007). It has been suggested that osteoclast precursors differentiate into active osteoclasts in response to two key factors (macrophage colony-stimulating factor [M-CSF] and RANKL). M-CSF induces the proliferation of bone marrow monocytes (BMMs) toward osteoclast precursors, sustains survival, and up-regulates expression of RANK, the receptor of RANKL (Suda et al., 2001). RANKL promotes osteoclast formation from osteoclast precursors in the presence of M-CSF (Arai et al., 1999; Koga et al., 2004). Binding of RANKL with its receptor, RANK, can induce the recruitment of TNF receptor-associated factor 6 (TRAF6), leading to the activation of NF-kB and mitogen-activated protein kinase (MAPK) pathways, and the subsequent up-regulation of transcription factor nuclear factor of activated T cells 1 (NFATc1) during osteoclastogenesis (Matsumoto et al., 2000; Wei et al., 2001; Asagiri and Takayanagi, 2007). NFATc1 then regulates a number of osteoclastogenesis-related marker genes, such as calcitonin receptor (CTR), matrix metalloproteinase 9 (MMP9), or cathepsin K, which lead to the formation of bone resorption pits during osteoclast differentiation (Takayanagi, 2007; Hwang and Putney, 2012). Targeted modulation of the NF-κB and MAPK signaling pathways to alter NFATc1 expression could be potentially useful in the prevention and treatment of bone erosion in RA.

Administration of recombinant human endostatin (rhEndostatin), which has characteristics of the native endostatin, has been shown to have an arthritis-inhibiting effect in adjuvant arthritis (Yue et al., 2007; Huang et al., 2014). A previous study showed that rhEndostatin treatment markedly suppresses secondary hind paw swelling and the polyarthritis index, as well as effectively inhibits bone degradation during adjuvant-induced arthritis (AA) in rats (Hu et al., 2012), suggesting that rhEndostatin has a strong protective effect against progression of AA in rats. Because bone loss in RA is principally caused by increased osteoclast activity, inhibiting osteoclastogenesis in mature osteoclasts is a promising approach for the prevention and treatment of bone resorption-related disorders (Zeng et al., 2016); however, whether or not rhEndostatin regulates osteoclast formation and function has not been reported. In this study we examined the effects of rhEndostatin on osteoclast formation and function *in vitro*, then elucidated the underlying molecular mechanisms.

## Materials and Methods

### Reagents and Antibodies

Fetal bovine serum (FBS) and α-modified essential medium (α-MEM) were purchased from Invitrogen (Carlsbad, CA, USA). Recombinant murine RANKL and M-CSF were purchased from Peprotech (London, UK). A tartrate-resistant acid phosphatase (TRAP) kit was obtained using a leukocyte acid phosphatase staining kit (Sigma–Aldrich Co., St. Louis, MO, USA). Antibodies against the non-phosphorylated and phosphorylated forms of p65, IκBα, ERK1/2, JNK, and p38 were obtained from Santa Cruz Biotechnology, Inc. (Santa Cruz, CA, USA).

### Animals and Ethics Approval

Experimental protocols were approved by the Ethics Committee of Shanghai Medical College of Fudan University (Permit: 20151201-001). All the animal experiments were made to minimize suffering and reduce the number of animals used. Male BALB/c mice (11 weeks old) were purchased from the Chinese Academy of Science (Shanghai, China). These mice were used to obtain osteoclast precursors from bone marrow cells.

### *In Vitro* Osteoclastogenesis Assay

Mouse bone marrow cells were obtained from male BALB/c mice (11 weeks old) which were euthanized using sodium pentobarbital. The tibias and femurs from mice were cut in half, and flushed with phenol red free α-MEM containing 10% charcoal-stripped FBS and 30 ng/mL of M-CSF. Cells were cultured in 25-cm^2^ tissue culture flasks at 37°C and 5% CO_2_. After 24 h, the non-adherent cells were used as osteoclast precursors and cultured for 3 days in the presence of M-CSF (30 ng/mL). After 3 days, the resulting adherent cells were used as bone marrow macrophages (BMMs). For differentiation into mature osteoclasts, osteoclast precursors were plated at a density of 1.5 × 10^6^ cells/well and cultured with M-CSF (30 ng/mL) and RANKL (100 ng/mL) in 24-well culture plates for 7 days with media replaced every 2 days (Wu et al., 2012; Baek et al., 2014).

### TRAP Staining

After 7 days in culture, the cells were fixed in 3.7% formaldehyde for 15 min at 4°C. Samples were stained for TRAP according to the manufacturer’s instructions. TRAP-positive multinuclear cells with greater than three nuclei were counted as osteoclasts under a light microscope.

### Cytotoxicity Assay

We detected the cytotoxic effect of rhEndostatin using a 3-(4,5-dimethylthiazol-2-yl)-2,5-diphenyltetrazolium bromide (MTT) kit (Beyotime Institute of Biotechnology, China), as previously described (Zhu et al., 2012).

### Pit Formation Assays

To measure resorption, cells were cultured and treated, as described above. Briefly, BMMs (2 × 10^5^ cells/well) were plated on bone slices and treated with rhEndostatin (50 mM) in the presence of M-CSF (30 ng/mL) and RANKL (100 ng/mL) for 7 days, then removed by sonication and the dentine slices (IDS, Boldon, UK) was stained with toluidine blue. The resorption areas were observed under a light microscope and analyzed with ImageJ software.

### Real-Time PCR Analysis

Real-time PCR was used to determine the effects of rhEndostatin treatment on the level of mRNA expression of RANKL-induced osteoclast specific genes (NFATc1, CTR, MMP9, and cathepsin K), as described previously (Zhu et al., 2012; Li et al., 2013). Gene-specific primers used in PCR are listed in Supplementary Table S1.

### Luciferase Reporter Assay

NF-κB transcriptional activity was measured using a luciferase reporter assay. Cells were transfected with 1 μg of pGL4.32[luc2P/NF-κB-RE/Hygro] vector and 1 μg of pGL4.44[luc2P/AP1 RE/Hygro] vector (Promega, Madison, WI, USA) using Lipofectamine 2000 (Invitrogen). To detect luciferase activity, the BMMs were lysed with 1 × passive lysis buffer (Promega) and the cell lysate was then mixed with luciferase assay substrate. Luciferase activity was measured using the dual-luciferase reporter assay system according to the manufacturer’s instructions (Promega).

### Western Blotting

Whole cells were lysed in lysis buffer, supplemented with protease inhibitor mixture (Roche Applied Science, Indianapolis, IN, USA). Fractionation of nuclear and cytosolic proteins was isolated using a Nuclear/Cytosol Fractionation Kit (BioVision, Inc., Mountain View, CA, USA), according to the manufacturer’s instructions. The lysates were fractionated on a 10% SDS-PAGE gel and transferred to PVDF membranes. The membranes were blocked for 1 h at room temperature with 5% skim milk and incubated with different antibodies overnight at 4°C. All of the primary antibodies were diluted to 1:800. After being rinsed, the membranes were incubated with secondary antibodies for 1 h at room temperature. The bands were visualized using film exposure with densitometry (Gel Logic 2200; Rochester, NY, USA). ImageJ software was used to analyze protein band intensity.

### Statistical Analysis

Data are expressed as the mean ± SEM. Statistical analysis was performed using SPSS 13.0 software. Statistical differences were assessed by one-way analysis of variance followed by a *post hoc* Tukey’s test. *P* < 0.05 were accepted as a significant difference.

## Results

### Effect of rhEndostatin on Osteoclast-Like Cell Formation

**FIGURE 1:**
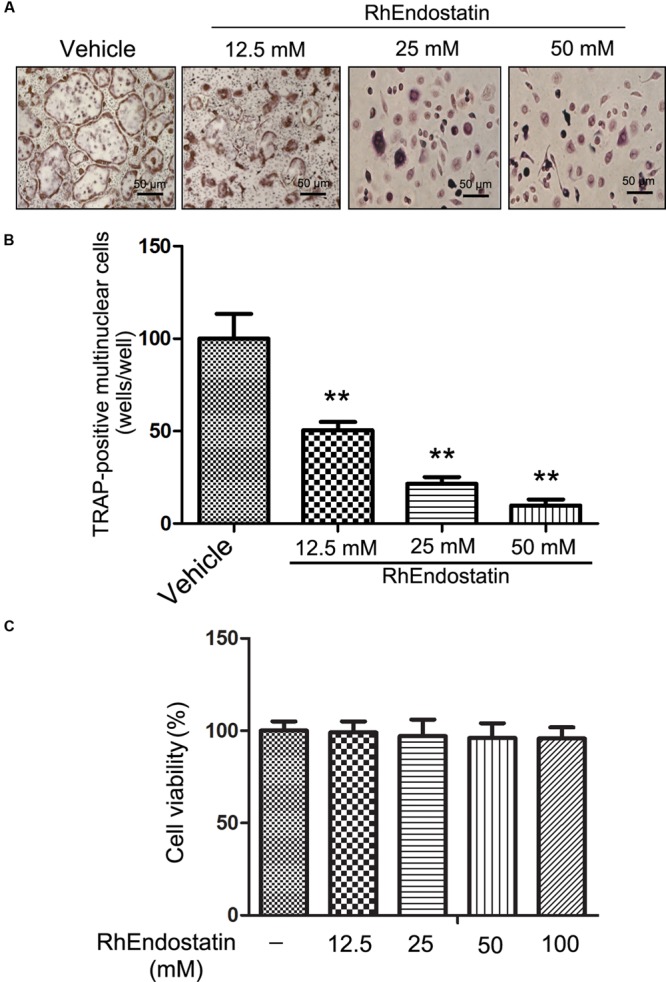
**Effects of rhEndostatin on RANKL-induced osteoclast-like cell formation in mouse BMMs. (A)** Mouse BMMs were cultured with vehicle (distilled water) or the indicated concentrations of rhEndostatin for 3 h, then stimulated with M-CSF (30 ng/mL) and RANKL (100 ng/mL) for 5 days. The osteoclasts were fixed and stained for TRAP staining. **(B)** TRAP-positive multi-nucleated cells with greater than three or more nuclei were counted as osteoclasts. **(C)** Effects of rhEndostatin on the cell viability of BMMs. BMMs were treated with the indicated concentrations of rhEndostatin for the indicated times. Cell viability was determined by the MTT method. ^∗∗^*P* < 0.01 compared with vehicle-treated control.

We first compared the inhibitory effects of rhEndostatin on osteoclast-like cell formation in mouse BMM cultures. Mouse BMMs were cultured in the presence of M-CSF and RANKL with increasing concentrations of rhEndostatin (12.5, 25, and 50 mM). As shown in **Figure 1A**, TRAP-positive multinuclear osteoclasts from BMMs formed within 3 days in control cultures. RhEndostatin inhibited osteoclast-like cell formation in a dose-dependent manner (**Figure 1B**). RhEndostatin (12.5, 25, and 50 mM) treatments produced a marked suppression of the number of osteoclasts derived from BMMs in a dose-dependent manner.

To determine whether or not cytotoxic effects of rhEndostatin contributed to suppressing osteoclast-like cell formation, we analyzed the cytotoxicity of rhEndostatin by MTT assay in BMMs (**Figure 1C**). rhEndostatin was minimally cytotoxic to BMMs at concentrations up to 100 μM, suggesting that the effects of rhEndostatin on osteoclast-like cell formation are not caused by cytotoxic effects of the compound.

### Effects of rhEndostatin on Stages of Osteoclast-Like Cell Formation

**FIGURE 2:**
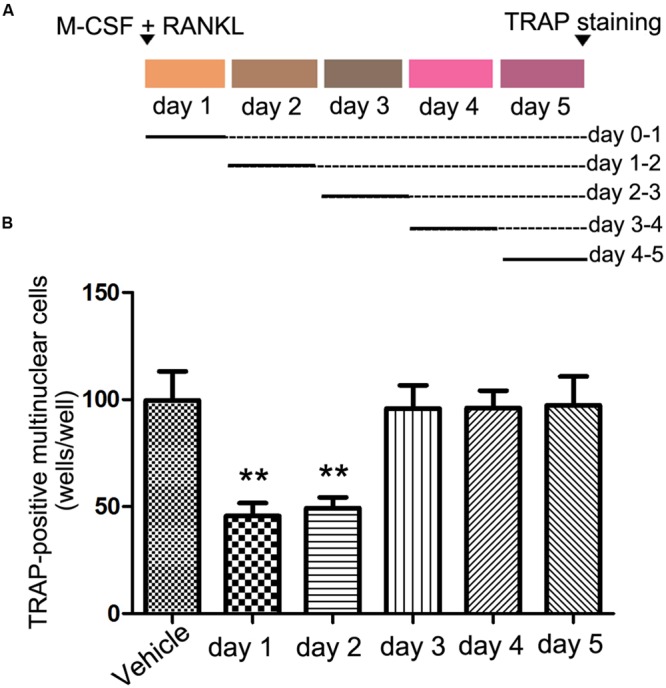
**Effects of rhEndostatin on osteoclast-like cell formation treated for different time periods. (A)** Schematic of the experimental design for the *in vitro* experiments. BMMs were cultured in the presence of M-CSF and RANKL for 5 days, and rhEndostatin (50 mM) was added to the BMM cultures at the indicated days. **(B)** The number of TRAP-positive multi-nucleated cells were counted. The data are presented as percentages relative to the vehicle-treated control group. ^∗∗^*P* < 0.01 compared with the vehicle-treated control.

Osteoclast formation requires some processes, including proliferation, differentiation, cell fusion, and multi-nucleation. To determine at which stage rhEndostatin inhibited osteoclast formation, rhEndostatin was added to BMMs (+M-CSF and RANKL) at different times during a 5-days culture period (**Figure 2A**). After exposure to rhEndostatin for 24 h, the culture media containing the rhEndostatin was washed off and changed to rhEndostatin-free culture media. The addition of rhEndostatin on days 0–1 and 1–2 markedly reduced osteoclast-like cell formation; however, rhEndostatin treatment at later stages (days 3–5) did not effectively block osteoclast-like cell formation. Our results demonstrated that rhEndostatin likely suppress osteoclast-like cell formation by targeting the early stage of differentiation (**Figure 2B**).

### Effects of rhEndostatin on the Bone Resorbing Function of Osteoclast-Like Cell Formation

**FIGURE 3:**
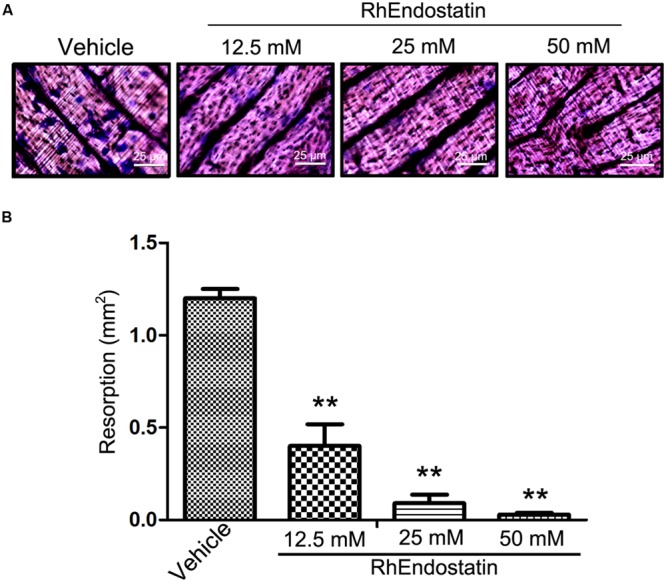
**Effects of rhEndostatin on bone resorbing function of osteoclasts of BMMs cells induced by M-CSF and RANKL. (A)** BMMs were seeded on dentine slices and treated with vehicle or rhEndostatin (12.5, 25, and 50 mM) for 3 h, then stimulated with M-CSF and RANKL. After culture for 5 days, the cells were removed from dentine slices, and the slices were stained with toluidine blue. **(B)** The total area of resorption pits was measured. ^∗∗^*P* < 0.01 compared with the vehicle-treated control.

To determine the functional implications of rhEndostatin inhibition of cytoskeletal organization, we assessed the capacity of osteoclast-like cells to resorb a mineralized matrix. BMMs were plated on bone slices and stimulated with M-CSF and RANKL for 5 days to generate mature osteoclasts in the presence or absence of rhEndostatin (12.5, 25, and 50 mM). As shown in **Figure 3A**, numerous bone resorption pits were formed on the dentine slices in control cultures. RhEndostatin (12.5, 25, and 50 mM) treatment strongly diminished areas of bone resorption pits resulting from M-CSF and RANKL-induced BMMs (**Figure 3B**). These findings demonstrated that rhEndostatin impaired osteoclast bone resorption *in vitro*.

### Effects of rhEndostatin on the Expression of Osteoclast-Specific Markers

**FIGURE 4:**
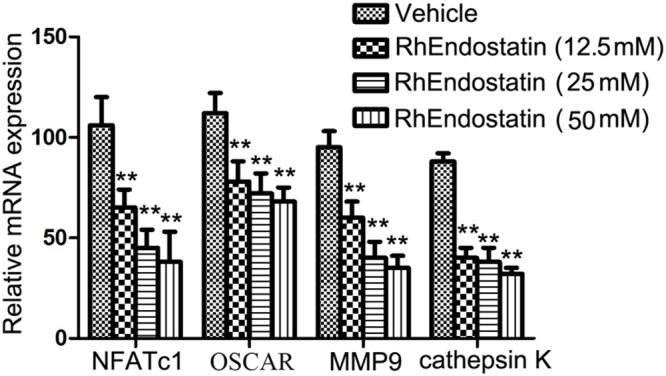
**Effects of rhEndostatin on the expression of osteoclast-specific markers.** BMMs were treated with vehicle or rhEndostatin (12.5, 25, and 50 mM) for 3 h, then stimulated with M-CSF and RANKL. After culture for 5 days, total RNA was extracted. Quantitative real-time qPCR was used to analyze the level of mRNA expression of the indicated genes. GAPDH was used as a loading control. ^∗∗^*P* < 0.01 compared with the vehicle-treated control.

During osteoclast formation and bone resorption, the level of expression of several specific genes, such as NFATc1, CTR, MMP9, and cathepsin K, are closely related to the eventual formation and function of osteoclasts (Takayanagi, 2007; Hwang and Putney, 2012). Therefore, in the present study we assessed the effects of rhEndostatin on the expression of these molecules in BMMs induced by M-CSF and RANKL. As shown in **Figure 4**, mouse BMMs were cultured in the presence of RANKL and M-CSF led to significant up-regulation of the expression of NFATc1, CTR, MMP9, and cathepsin K mRNA in control cultures. Moreover, rhEndostatin treatment dramatically reduced the levels of NFATc1, CTR, MMP9, and cathepsin K mRNA in a concentration-dependent manner. Our results suggested that rhEndostatin suppresses the formation and function of osteoclasts by inhibiting the gene expression of osteoclast-specific markers.

### RhEndostatin Inhibited the Activation of the NF-κB Pathway by Inhibiting IκBα Phosphorylation and the Translocation of Cytosolic NF-κB-p65 to the Nucleus

**FIGURE 5:**
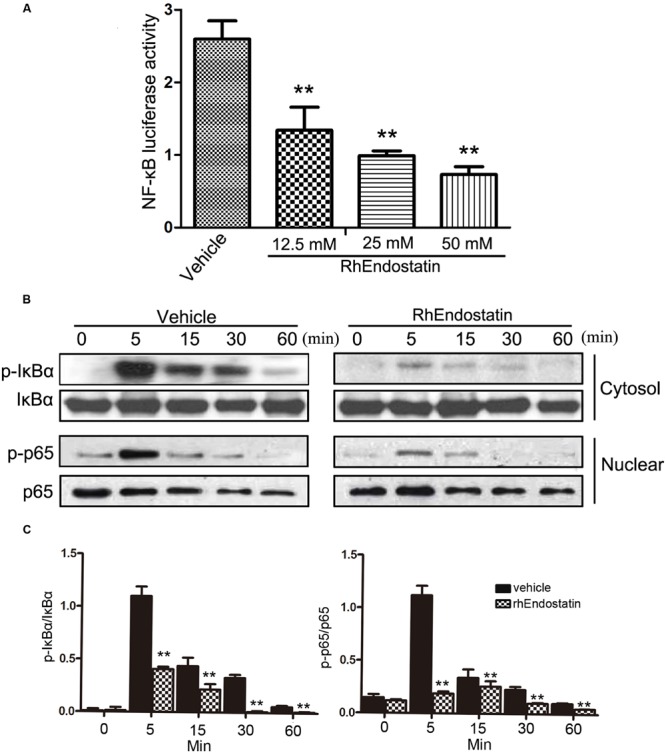
**Effect of rhEndostatin on RANKL-induced activation of NF-κB in BMMs. (A)** BMMs were transiently co-transfected with NF-κB luciferase reporter gene constructs, then pre-treated with vehicle or the rhEndostatin (12.5, 25, and 50 mM) for 3 h. RANKL was added and cells were incubated for an additional 12 h. The cells were harvested and the level of NF-κB activation was determined using the luciferase reporter assay system. ^∗∗^*P* < 0.01 compared with the vehicle-treated control **(B)** BMMs were pre-treated with vehicle or the rhEndostatin (50 mM) for 3 h, then stimulated with RANKL (100 ng/mL) for the indicated times. The cytosolic level of phosphorylated NF-κB IκBα and the nuclear phosphorylation of p65 levels were determined by western blot analysis. **(C)** Quantification of phosphorylated NF-κB IκBα and phosphorylation of p65 protein expression were normalized by total IκBα and p65, respectively. ^∗∗^*P* < 0.01 compared with the vehicle-treated control.

Receptor activator of NF-κB ligand-induced NF-κB is an essential transcription factor that is vital to osteoclast differentiation. Mice that lack the NF-κB p50 and p52 subunits fail to generate mature osteoclasts, which leads to the development of severe osteopetrosis (Asagiri and Takayanagi, 2007). The activation of NF-κB is regulated by three major steps, as follows: IκBα phosphorylation; degradation of IκBα; and nuclear translocation of the p65 subunit of NF-κB. We determined whether or not rhEndostatin affects RANKL-induced NF-κB activation. Osteoclast formation was induced using BMMs in RANKL in the presence or absence of rhEndostatin. RANKL-induced NF-κB activation was assessed using an NF-κB luciferase reporter gene assay. As shown in **Figure 5A**, RANKL stimulation dramatically increased NF-κB activity. Indeed, rhEndostatin attenuated RANKL-induced NF-κB activity in a dose-dependent manner. The cytosolic level of phosphorylation of IκBα was detected by western blot, and we showed that RANKL induced the cytosolic level of phosphorylation of IκBα reached the maximum level after 5 min, whereas the cytosolic level of phosphorylation of IκBα was markedly decreased by pre-treatment with rhEndostatin in BMMs (**Figures 5B,C**). To determine whether or not rhEndostatin inhibited RANKL-induced nuclear translocation of p65, nuclear proteins were extracted at 0, 5, 15, 30, and 60 min, and western blot analysis was carried out in BMMs. As shown in **Figures 5B,C**, treatment with RANKL significantly increased the nuclear phosphorylation of NF-κB p65, while rhEndostatin attenuated the RANKL-induced increase in nuclear NF-κB p65 phosphorylation. In contrast, p65 in cytoplasmic extracts dramatically increased treatment by rhEndostatin (Supplementary Figure S1). Thus, these data indicate that rhEndostatin inhibited RANKL-induced osteoclast formation by regulating the activation of NF-κB.

### RhEndostatin Inhibits the RANKL-Induced Phosphorylation of MAPKs

**FIGURE 6:**
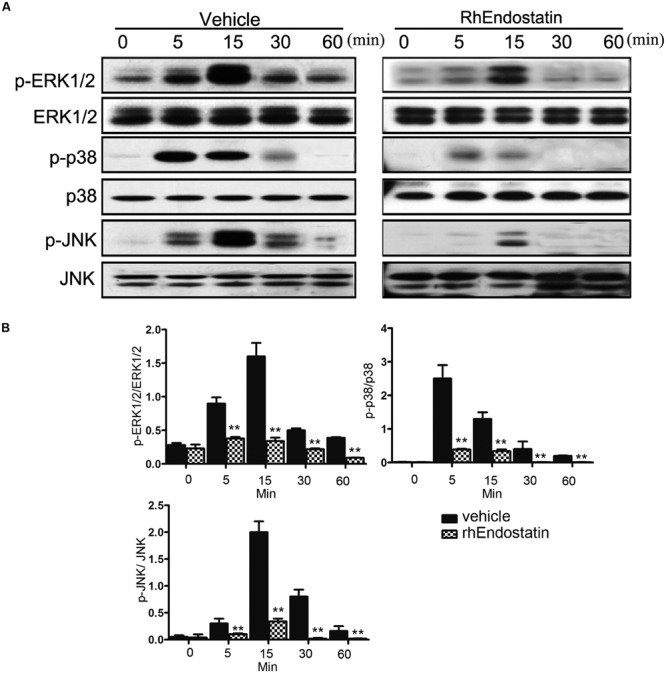
**Effect of rhEndostatin on RANKL-induced activation of MAPK in BMMs. (A)** BMMs were pre-treated with vehicle or rhEndostatin (50 mM) for 3 h, then stimulated with RANKL (100 ng/mL) for the indicated times. Total protein was extracted and analyzed by western blot analysis using antibodies against phospho-ERK1/2, ERK1/2, phospho-JNK, JNK, phospho-p38, and p38. **(B)** Quantification of phosphorylated ERK1/2, p38, and JNK protein expression was normalized by total ERK1/2, p38, and JNK expression, respectively. ^∗∗^*P* < 0.01 compared with the vehicle-treated control.

In addition to the NF-κB signaling pathway, activation of the MAPKs plays an important role in osteoclast formation (Huang et al., 2006). To evaluate the effects of rhEndostatin on the MAPKs following stimulation with RANKL in BMMs, we examined the activation of three well-established MAPK sub-families (ERK1/2, JNK, and p38 MAPK) by western blot. As shown in **Figures 6A,B**, RANKL-induced phosphorylation of ERK1/2 and JNK reached a maximum at 15 min. Moreover, RANKL-induced phosphorylation of p38 reached a maximum at 5 min; treatment of BMMs with 50 mM rhEndostatin significantly suppressed phosphorylation of ERK, p38, and JNK (**Figures 6A,B**). Taken together, our results suggested that rhEndostatin suppresses osteoclastogenesis by attenuating the activation of MAPK pathways induced by RANKL.

## Discussion

The present study demonstrated that rhEndostatin inhibits osteoclast-like cell formation and osteoclast differentiation *in vitro* in the initial stage. At the molecular level, rhEndostatin inhibited RANKL-induced NF-κB activation by affecting the phosphorylation and degradation of IκBα and NF-κB p65 nuclear translocation, as well as activation of the MAPK signaling pathways.

Osteoclasts are unique multinuclear giant cells formed by the fusion of precursor cells of the monocyte/macrophage lineage (Li et al., 2013). It is known that M-CSF induces osteoclast precursor cell proliferation into the osteoclast lineage, whereas RANKL stimulates subsequent osteoclast precursor differentiation into osteoclasts (Asagiri and Takayanagi, 2007; Kim et al., 2014). Osteoclast differentiation from precursors can be induced by the presence of RANKL, a key regulator of osteoclast formation. Up-regulation of RANKL expression is produced by fibroblast-like synoviocytes in RA (Kim et al., 2011). As shown herein, rhEndostatin significantly reduced the number of TRAP-positive multinuclear osteoclasts derived from BMMs in the presence of RANKL and M-CSF, suggesting that rhEndostatin inhibits osteoclast formation. It is evident that the effect of rhEndostatin is restricted to the early stage of osteoclastogenesis because delayed addition of rhEndostatin did not affect osteoclast formation. It is widely believed that the formation of osteoclastic bone resorption pits arises in the conjunction area with the process of osteoclast differentiation (Zou et al., 2007). In the current study, M-CSF and RANKL stimulation in BMMs led to the formation of many resorption pits on dentine slices, and rhEndostatin treatment significantly diminished areas of bone resorption pits resulting from M-CSF and RANKL-induced BMMs. Moreover, the expression of osteoclast specific genes, such as NFATc1, CTR, MMP9, and cathepsin K, was reduced in the presence of rhEndostatin; however, multinucleated cell activation is required for the activity of osteoclasts. Thus, cytoskeletal arrangements can be established for the purpose of adherence to the mineralized surface, as can be noted on administration of some bisphosphonates, such as sodium alendronate, to animals or on the addition of these bisphosphonates to cultures (Arana-Chavez and Bradaschia-Correa, 2009). Following treatment with alendronate, osteoclast precursors are recruited, proliferate, and undergo differentiation to multinucleated osteoclasts, but do not undergo activation. For this reason, resorptive activity is negatively affected (Bradaschia-Correa et al., 2013). Therefore, a focus area for future research is to determine whether or not rhEndostatin directly affects the activity of osteoclasts.

As a central mediator of immune and inflammatory responses, NF-κB activation is also thought to be one of the critical transcription factors of RANKL-induced osteoclast formation and bone resorption. Gene knockout studies demonstrate a key role of NF-κB signaling in osteoclast formation, including p50 and p52 subunits of NF-κB double-knockout mice that manifested severe osteopetrosis due to impaired osteoclast formation (Novack, 2011). The current results showed that 50 mM rhEndostatin treatment inhibited the activation of NF-κB by inhibiting the phosphorylation of IκBα, thereby blocking the nuclear translocation and activation of the p65 subunit of NF-κB. In particular, there was no significant difference in cellular proliferation at 25 and 50 mM rhEndostatin compared with controls. Therefore, 50 mM rhEndostatin was used for all subsequent experiments in molecular assays.

Three major subfamilies of MAPKs (ERK1/2, JNK, and p38) shown to be key regulators of various cellular responses, including cell migration, apoptosis, differentiation, and proliferation (Krens et al., 2006). These kinases also play an important role in the differentiation and activation of osteoclasts and have been considered as key molecular targets associated with inflammatory bone destruction in RA (Mbalaviele et al., 2006). Herein we found that the RANKL-induced phosphorylation of ERK1/2, JNK, and p38 decreased in response to rhEndostatin treatment in murine BMMs, suggesting that inhibition of the MAPK pathway is possible in the inhibitory action of this agent.

Although the RANKL/RANK axis has undoubtedly been considered to be an essential factor for osteoclastogenesis that occurs in the bone remodeling physiologic process, tumor necrosis family superfamily members are known to influence osteoclast formation and bone resorption by a RANKL-independent mechanism (Sabokbar et al., 2015; Yuan et al., 2015). Further consideration should take into account the fact that rhEndostatin presents similar effects over pro-inflammatory cytokines involved in RA pathology in addition to RANKL.

To the best of our knowledge, the current study is the first to provide evidence that rhEndostatin inhibits osteoclast formation and bone resorption *in vitro*. Moreover, we have described the underlying mechanism of action through which rhEndostatin negatively regulates osteoclast formation by suppressing NF-κB and MAPK signaling pathways. These results suggest that rhEndostatin could be an attractive therapeutic agent for the treatment of osteoclast formation and bone resorption in the arthritic joint. Although our data demonstrates that rhEndostatin inhibits the formation and function of osteoclasts *in vitro*, further studies are still warranted to comprehensively explore the role of endostatin in human osteoclast progenitors and patients with RAs.

## Author Contributions

NC, F-LY, and M-DZ contributed to the conception and design of the study, the acquisition of data, and the analysis and interpretation of the data. All authors contributed to the acquisition of data and the analysis and interpretation of the data. All authors participated in drafting or revising the manuscript, and all authors approved the final version of the manuscript for submission.

## Conflict of Interest Statement

The authors declare that the research was conducted in the absence of any commercial or financial relationships that could be construed as a potential conflict of interest.
