# Supplementary material for: Recombinant Human Endostatin Suppresses Mouse Osteoclast Formation by Inhibiting the NF-κB and MAPKs Signaling Pathways
Source: Front Pharmacol. 2016 Jun 1;7:145. doi: 10.3389/fphar.2016.00145 (PMC4887464; doi:10.3389/fphar.2016.00145)
Supplement: Supplementary file 1 [file Data_Sheet_1.DOCX]

**Table S1. Primer Sequences for PCR**

| NFATc1 | CGGGAAGAAGATGGTGCTGT | TTGGACGGGGCTGGTTAT |
| --- | --- | --- |
| CTR | CGGACTTTGACACAGCAGAA | AGCAGCAATCGACAAGGAGT |
| MMP9 | TGGGGGGCAACTCGGC | GGAATGATCTAAGCCCAG |
| Cathepsin K | CAGCAGAGGTGTGTACTATG | GCGTTGTTCTTACTTCGAGC |

**Supplementary Figure 1**


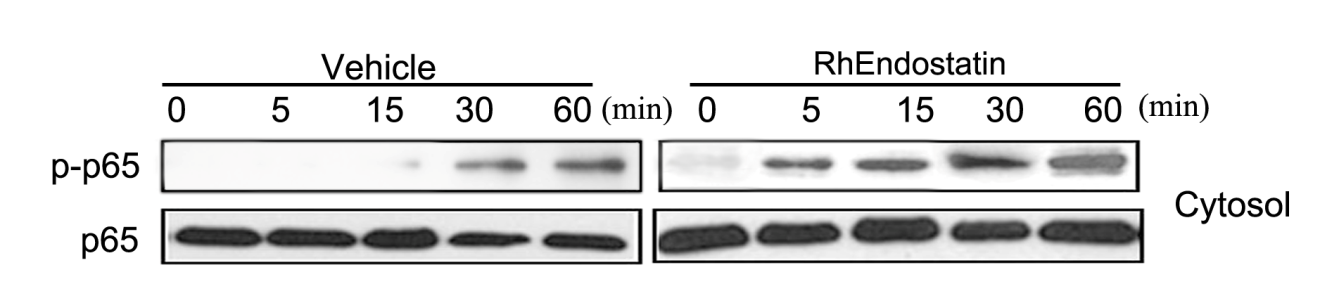


BMMs were pre-treated with vehicle or the rhEndostatin (50 mM) for 3 h, then stimulated with RANKL (100 ng/mL) for the indicated times. The cytosolic level of phosphorylated p65 levels were determined by western blot analysis.
